# Effectiveness of community health workers in improving early initiation and exclusive breastfeeding rates in a low‐resource setting: A cluster‐randomized longitudinal study

**DOI:** 10.1002/fsn3.1559

**Published:** 2020-04-28

**Authors:** Dorothy Mituki‐Mungiria, Prisca Tuitoek, Aniko Varpolatai, Douglas Ngotho, Elizabeth Kimani‐Murage

**Affiliations:** ^1^ Department of Human Nutrition Egerton University Egerton Kenya; ^2^ Muranga University College Muranga Kenya; ^3^ School of Education University of Western Ontario London ON Canada; ^4^ Faculty of Health Sciences Egerton University Egerton Kenya; ^5^ African Population and Health Research Center Nairobi Kenya

**Keywords:** breastfeeding initiation, community health workers, exclusive breastfeeding

## Abstract

Little evidence exists in Kenya on the potential of community health workers (CHWs) in promoting exclusive breastfeeding (EBF) and early breastfeeding initiation (EBI) in resource‐restricted settings where very low EBF rates (2% to 12%) have been documented. The study utilized CHWs and assessed their effectiveness in promoting EBF and EBI. The cluster‐randomized longitudinal design was used and sixteen villages from Kiandutu Slum in Thika randomly assigned into either intervention group (IG) or comparison group (CG). Pregnant women attending Maternal Child Health (MCH) clinic were recruited. The IG received nutrition education sessions conducted by CHWs at home, two prenatally and six postnatally, plus the routine MCH care. The CG went through routine MCH care only. Infants feeding data were collected at 6, 10, 14, and 24 weeks postpartum by research assistants blinded to the intervention allocation. Differences in EBF and EBI in the two groups were tested using *χ* ^2^ tests, Kaplan–Meier survival analysis and generalized estimating equations. Of the 526 recruited in the study, 431 remained and were included in the analysis (IG = 176) and CG (225). The prevalence of EBF at 24 weeks was 45.3% in the IG compared with 15.0% in the CG, revealing a statistically significant difference log rank = 20.277, (1, *n* = 314) *p* < .001. The difference was not statistically significant in EBI prevalence between the IG (58.2%) and CG (60.3%; *χ* ^2^ = 0.008, *p* = .928). The CHWs have potential effectiveness in promoting EBF but not EBI. The link between the health center and CHWs should be strengthened to promote EBF.

## Graphical abstract

The study utilized Community Health Workers (CHWs) and assessed their effectiveness in promoting Exclusive Breastfeeding and breastfeeding initiation in a cluster‐randomized longitudinal study. The CHWs have potential effectiveness in promoting EBF they should be engaged and empowered by County Governments in the promotion of EBF particularly in the resource‐restricted settings.

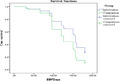

## 1 INTRODUCTION

Breastfeeding has the single largest potential impact on child survival among all preventive interventions (Bhandari, Kabir, & Salam, 2008; Du Plessis, 2009; WHO, 2008) due to immunity factors found in breast milk. It protects infants from diarrhea, respiratory infections, and other diseases, (Horta & Victora, 2013; WHO, 2009). Breast milk also contains all nutrients required by the infant (Kramer, 2008; Nielsen, Reilly, Fewtrell, Eaton, & Wellz, 2011). Furthermore, some studies have linked suboptimal breastfeeding with chronic diseases (Horta, Bahl, Martines, & Victora, 2007; Lawn, Kerber, Enweronu‐Laryea, & Bateman, 2009). Other benefits of breastfeeding include improved cognitive development, higher school attainment, higher intelligent quotient (IQ), and increased economic productivity later in life (Victora et al., 2015, 2016). The World Health Organization (WHO) recommends optimal breastfeeding which includes; early initiation of breastfeeding (within 1 hr of birth); EBF for the first six months and continued breastfeeding up to two years of life (WHO, 2003). Several studies suggest a cause‐effect relationship between early breastfeeding initiation (EBI) and reduction in infection‐specific neonatal mortality (Biks, Berhane, Worku, & Gete, 2015; Edmond, Kirkwood, Amenga‐Etego, Owusu‐Agyei, & Hurt, 2007; Edmond et al., 2006; Mullany et al., 2008). Late initiation of breastfeeding denies infants the maximum benefits of colostrum which is critical in boosting an infant's immunity and encouraging the passage of the first stool. Globally, rates of EBI and EBF are generally low (Black et al., 2013). EBI rates are 62% (KNBS & ICF Macro, 2014) similar to the rates of exclusive breastfeeding which stands 61%. Rates of EBF in the resource‐restricted settings are very low at 2% (Kimani‐Murage et al., 2011). Under nutrition including suboptimal breastfeeding is associated with 3.1 million global child deaths annually (Black et al., 2013). Breastfeeding promotion is therefore pivotal to infant health promotion and survival especially in the low‐ and middle‐income countries (LMICS). Many health promotion interventions have utilized Community Health Workers (CHWs) in their programs. The CHWs were identified as a distinguishing feature for the provision of primary health care for people in resource‐restricted settings in the Alma‐Ata Declaration (Lehmann & Sanders, 2007). They act as a mitigating factor where healthcare access is limited due to limited numbers of healthcare workers by providing essential Maternal Child Health (MCH) care at the household and community level. They also provide education, preventive health services, and play the role of liaison between the community and facility‐based services (Bhutta, Lassi, Pariyo, & Huicho, 2010; UNFPA, 2011). The use of CHWs, compared to usual healthcare services, has been found to be effective in increasing rates of early initiation of breastfeeding (within one hour); rates of breastfeeding and EBF (Lewin et al., 2010). Findings from interventions that were implemented to promote EBF using CHWs indicate effectiveness (Balaluka et al., 2012; Gilmore & McAuliffe, 2013; Tylleskar et al., 2011), although Kimani‐Murage et al. (2015) found no difference in the intervention (that utilized CHWs to promote EBF) and control groups (that received usual care). Evidence of the effectiveness of CHWs in Kenya is therefore scanty. The present study examines the efficiency of CHWs in promoting early initiation and EBF among mothers in the context of a cluster‐randomized controlled nutrition education intervention in a low‐resource urban setting. Rates of EBF in these settings have been found to be extremely low (Kimani‐Murage et al., 2011; Ochola, Labadarios, & Nduati, 2012).

## 2 METHODS AND MATERIALS

### 2.1 Study site

The study was conducted in Kiandutu slum in Kiambu County‐Thika West District. Kiandutu slum is one (1) kilometer from Thika town, which is about 40 km Northeast of Nairobi. The informal settlement has 17 villages each with a village elder (Kariuki, 2012) and is served by two health centers, Kiandutu and Makongeni.

### 2.2 Study design and sample size calculation

The study was a cluster‐randomized longitudinal study conducted between September 2013 and October 2014. Cluster randomization design is used to evaluate interventions implemented at the community level, for logistical convenience and to avoid infiltration (Hayes & Bennet, 1999) hence the design was found suitable for this study. The study entailed two study groups the IG and the CG. To obtain a representative sample for the two groups, a level of precision of 5% (for a two‐sided *t* test) and power of 80% was set. The sample was multiplied by a design effect of 2 and by 20% for general attrition and 6% loss from low birth weight (LBW). The formula gave a requirement of 157 per arm but this figure was inflated by 60% to cater for high mobility characteristic of the slum observed during the pilot study. A total of 526 mothers were recruited after informed consent.

### 2.3 Randomization and sampling

**FIGURE 1:**
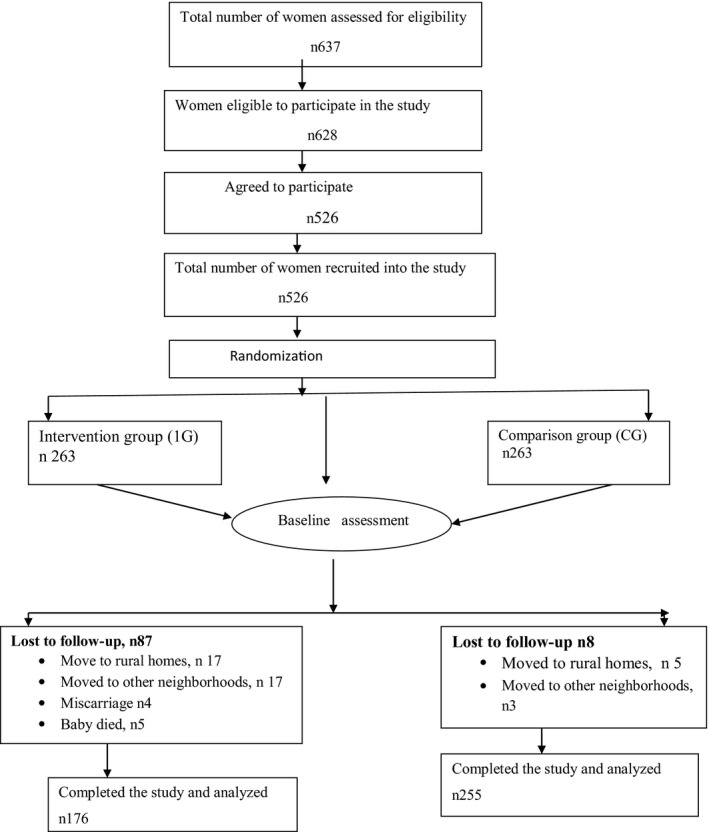
Schematic representation of the recruitment process for the study participants

The Microsoft^®^ excel function was used to randomize sixteen out of the seventeen villages into either intervention or comparison groups. The principle investigator recruited pregnant women attending prenatal clinic in Kiandutu and Makongeni health centers based on the village of residence. A two‐stage screening process was applied in sampling. Initial screening of pregnant women took place during the first prenatal visit. The second screening took place during the first postnatal visit. The inclusion criteria for the initial screening included being over 18 years less than six (<6) months gestation; without a history of chronic disorders such as hypertension, diabetes, HIV, and tuberculosis. Postnatal inclusion criteria included; term delivery, singleton births, and a birth outcome more than 2.5 kg. The recruitment of the women took place between September 2013 and December 2013 and was based on informed written consent (by signature or thump print). The recruitment took place on a rolling basis until the desired sample size of 263 per arm was attained. Figure 1 is the schematic representation of the randomization procedure.

### 2.4 Selection and training of the research team

Four CHWs, residents of the area, with a minimum of secondary level of education and two nutritionists with undergraduate‐level education were recruited and trained on Mother Infant and Young Child Nutrition (MIYCN) using the national MIYCN guidelines (2013). The 3‐day training was conducted by a Ministry of Health Nutritionist (also trained on MIYCN). A practical session was facilitated on one afternoon following the training at the Thika Level 5 general hospital. Two enumerators, graduates in nutrition, fluent in Kiswahili and English were selected for the study and trained for 3 days. The training content was based on discussions on the following topics: the purpose of the study; a review of interviewing techniques; providing explanatory information on items in the research questionnaires and how to record responses on the research tool systematically. Standardization of the data collecting tools and procedures for both the researcher and the enumerators was accomplished through group sessions by mimicking the respondent researcher situation. The study used validated semi‐structured questionnaires.

### 2.5 Description of the intervention

The intervention emphasized the (a) importance of EBF and EBI (b) conditions infants were susceptible to with early complementary feeding (c) developing breastfeeding confidence (d) good dietary practices for mothers and (e) proper attachment. The study nutritionists educated mothers (twice prenatally) in the intervention group at the health centers to create a link between the CHWs and the health center. The CHWs educated participants in the IG during home visits, (two times before delivery, during the first week of delivery and thereafter every month until the sixth month postpartum) in addition to routine clinic care. The mothers in the IG also received breastfeeding support from the CHWs. The CHWs helped solve breastfeeding problems, guided mothers on positioning techniques during breastfeeding and continually encouraged them to EBF their infants up to six months. Mothers in the CG received only the usual routine care from the health facilities. These included group counseling on dietary diversification, family planning, good hygiene, and the importance of EBF for the first 6 months done during the first clinic visit after delivery. The counseling was, however, inconsistent due to time constrains reported by healthcare providers at the health centers.

### 2.6 Data collection

To ensure content, semantic and technical equivalence were ascertained, the English version of the semi‐structured questionnaire was translated into Kiswahili with the help of a Linguist from Egerton University. The Kiswahili questionnaires were then translated into English through blind back‐translation by a neutral person. Adjustments were made by the research team. The questionnaire was pilot tested as documented in Mituki et al. (2017). The primary outcome of the study was the prevalence of EBF and was calculated as the ratio of infants at a particular postpartum visit (6 weeks, 10 weeks 14 weeks & 6 months) who were fed on breast milk alone in the 24‐hr preceding the interview to the total number of infants in the specific groups (intervention or comparison) and the total number of infants under the study. Infants who did not receive any other food or liquids other than breast milk were deemed to be exclusively breastfed. The cumulative prevalence of EBF was also determined at 6 months. The secondary outcome of the study EBI was determined by asking mothers how much time had elapsed before breastfeeding was initiated for the index infant. The prevalence of timely initiation of breastfeeding was then calculated as the ratio of infants’ breastfeed within 1 hr of delivery to the total number of infants.

### 2.7 Statistical analysis

Data were analyzed using the Statistical Package for Social Sciences (SPSS) computer program version 20 (IBM SPSS Statistics, IBM Corp.). Continuous variables were described by means and ranges. Categorical data were described in terms of numbers and percentages. Descriptive statistics, *χ* ^2^ test (adjusted for cluster randomization), generalized estimating equations (GEE), and Kaplan–Meier survival analysis were used to compare variables in the two study groups. All tests were two‐sided and were considered statistically significant at *p* < .05.

## 3 RESULTS

A total of the 628 women who met the study inclusion criteria were invited to participate in the study, 526 consented to participate and were enrolled into the study. Ninety‐two declined, 80% (74) without any reason, twelve (12) cited intention to return to their rural homes, while 6 cited lack of time. The 526 study participants were randomized into either the IG (263) or the CG (263). Eighty‐seven (33%) from the IG were lost to follow‐up while only eight (3%) in the CG.

### 3.1 Socio‐demographic characteristics of the study participants

Table 1 summarizes the socio‐demographic characteristics of the respondents at the baseline. The overall mean age was 24.06 ± 4.38 years old (range 18 to 38 years) intergroup means were 24.15 ± 4.36 and 23.99 ± 4.41years in the IG and CG respectively and were not statistically different (*p* > .05). Mothers were predominantly from the Kikuyu community (59.1%), with no statistically significant difference (*p* > .05) between the IG (57%) and CG (61.2%). Majority (89.5%) were married, and almost evenly split between primiparas (44.9%) and multiparas (54.8%). Furthermore, results in Table 1 indicate that majority (69.4%) of women had above primary education and the difference in education by group was statistically insignificant by Pearson chi‐square (*p* = .395). Household income was categorized into <11,700 and above 11,700 monthly, Kenya shilling (KES). Households with below KES 11,700 were 44.9% (44.2% and 45.7% in the IG and CG, respectively) while those above 11,700 KES were 55.3%. (56.3% and 54.3% in the IG and CG, respectively (1 US $ is equivalent to 100 KES). Between‐group differences were statistically insignificant by Fisher's exact test (*p* = .17).

**TABLE 1:** Percentage distribution of socio‐economic and demographic characteristics of the study participants

	Intervention (*n* = 263; %)	Comparison (*n* = 263; %)	All (*n* = 526; %)	*χ* ^2*^	*p*‐Value^*^
Ethnicity					
Kikuyu	57.0	61.2	59.1	5.344	.501
Kamba	23.2	16.3	19.8		
Luyha/Kisii	5.7	8.4	7.0		
Luo	4.9	5.7	5.3		
Meru	3.0	3.4	3.2		
Embu	3.8	3.0	3.4		
Others	2.3	1.9	2.1		
Marital status					
Married	88.2	90.9	89.5	0.995	.319
Single/Separated/Divorced	11.8	9.1	10.5		
Type of employment					
Informal	22.4	24.3	23.4	1.549	.671
Formal	6.8	4.2	5.5		
Business	22.8	22.4	22.6		
None	47.9	49	48.5		
Parity					
Primi‐parous	54.4	55.1	54.8	0.031	.861
Multi‐parous	45.6	44.9	45.2		
Education level					
≤Primary	28.9	32.3	30.6	0.725	.395
>Primary	71.1	67.7	69.4		
Household income					
≤11,700	44.2	45.7	44.9	3.568	.708
>11,700	56.3	54.3	55.3		

Note

Comparison between intervention and comparison group using chi‐square test; significance level, *p* < .05.

### 3.2 Effectiveness of community health workers

#### 3.2.1 Prevalence of early initiation of breastfeeding

Prevalence of EBI was determined by categorizing the mothers who initiated breastfeeding in less than an hour and over one hour after delivery. Thus, the prevalence of EBI (<1 hr) was 58.2% among mothers in the IG and 60.3% for those in CG, the difference was statistically insignificant with adjusted *χ* ^2^ test (*χ* ^2^ = 0.008, *p* = .928).

#### 3.2.2 Prevalence of EBF at 6, 10, 14 weeks, and 6 months

At the 6th week, a higher percentage of mothers in the IG (89.3%) compared to the CG (81.6%) were exclusively breastfeeding; however, the difference was statistically insignificant with adjusted *χ* ^2^ test, (*χ* ^2^ = 2.000, *p* = .157). At the 10th week, the mothers in the IG were more likely to exclusively breastfeed (80.3%) compared with 71.7% mothers in CG. The difference was, however, not statistically significant (*χ* ^2^ = 1.668, *p* = .196). At 14 weeks, a higher percentage of mothers in the IG (64.3%) were exclusively breastfeeding compared with those in the CG (43.5%). The difference was still statistically insignificant with adjusted *χ* ^2^ test (*χ* ^2^ = 7.395, *p* = .065). Similarly at the 6th month, mothers in the IG were more likely to exclusively breastfeed (45.3%) compared with those in the CG (15.0%), this time with a statistically significant difference even with an adjusted *χ* ^2^ test (*χ* ^2^ = 17.725, *p* = .000).

#### 3.2.3 Cumulative rate of EBF since birth

At six months, the cumulative percentage of mothers exclusively breastfeeding was 38.1% for the IG compared to 13.1% for the CG, with a statistically significant difference (*χ* ^2^ = 28.198, *p* = .010).

**FIGURE 2:**
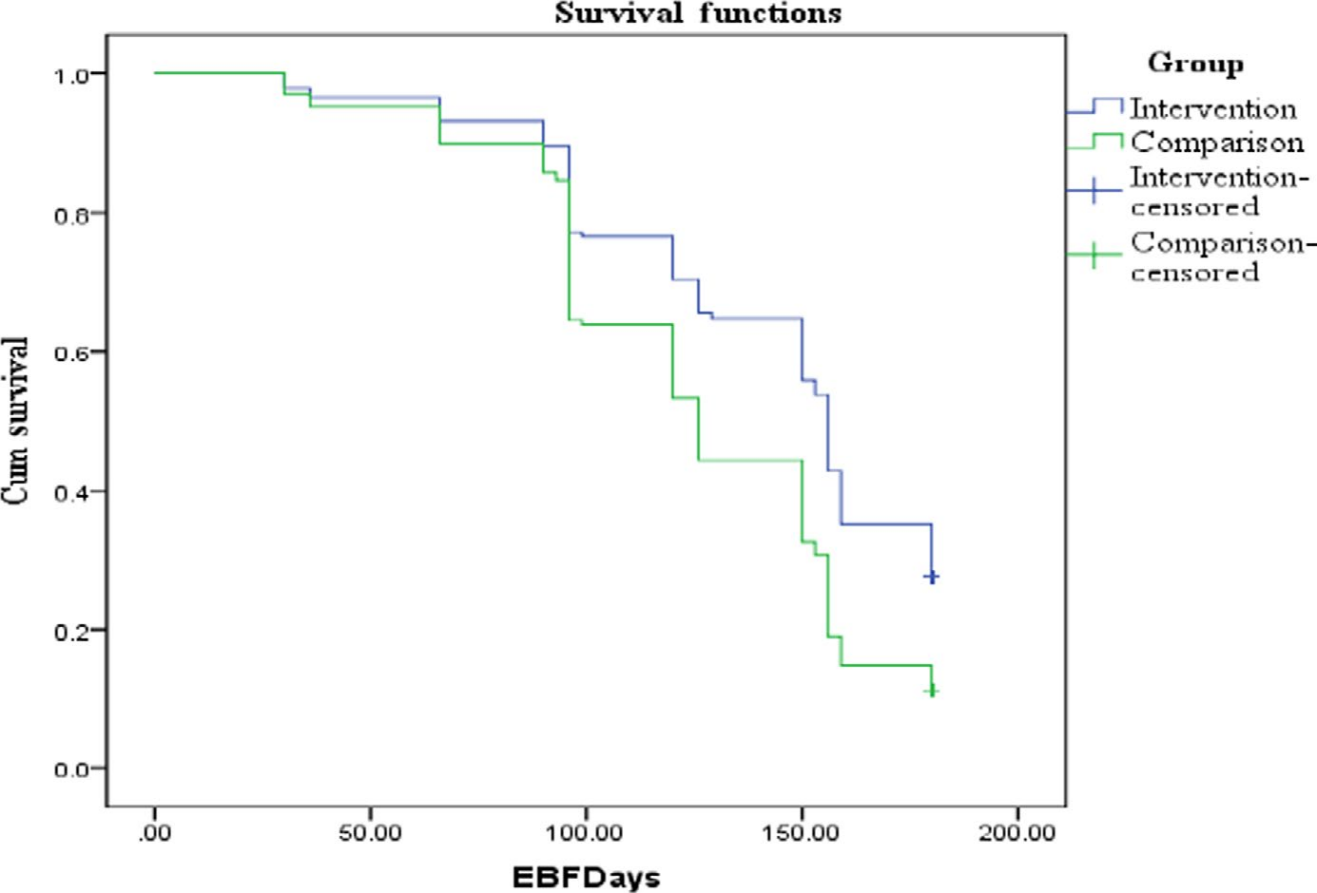
Kaplan–Meier survival analysis for duration of EBF of comparison versus intervention groups in days

The overall mean of EBF was 133.653 days (±2.279) by Kaplan–Meier survival analysis, (Figure 2) with a mean duration of 142.717 days in the IG (±3.322) verses 125.876 days (±3.017) in the CG. A log rank test was run to determine if there were statistically significant differences in the survival distribution for the two groups, and a significant difference was found, log rank 20.277, (1, *n* = 314) *p* < .001).

Analysis using generalized estimating equation (GEE) was further carried out to find out if this effect persisted after controlling for factors that had been found to be significantly associated with EBF at the univariate level. The factors included into the GEE model were as follows: time (data were collected at baseline, mid and end of intervention), breastfeeding self‐efficacy (BSES), perceptions toward exclusive breastfeeding, household income, hemoglobin (HB) levels, and household food security. The effect persisted (Table 2), with a significance of 0.001 (log odds, 10.32 at 95% CL of 4.26–16.39).

**TABLE 2:** Effectiveness of CHWs delivering nutrition education for promotion of EBF and EBI

Parameter	*B*	Std. error	95% Wald confidence interval		Hypothesis test		
			Lower	Upper	Wald Chi‐square	*df*	Sig.
(Intercept)	85.75	17.95	50.5	120.93	22.82	1	0.001*
[Time = 1.00]	2.72	1.91	−1.01	6.46	2.04	1	0.153
[Time = 2.00]	−5.98	1.77	−9.46	−2.51	11.39	1	0.001*
[Time = 3.00]	0^a^	.	.	.	.	.	.
[Group = 1]	10.32	3.10	4.26	16.39	11.12	1	0.001*
[Group = 2]	0^a^	.	.	.	.	.	.
[Edu_Mother = 1]	−13.76	3.47	−20.57	−6.95	15.70	1	0.001*
[Edu_Mother = 2]	0^a^	.	.	.	.	.	.
[Income = 1]	0.612	2.87	−5.01	6.23	0.046	1	0.831
[Income = 2]	0^a^	.	.	.	.	.	.
[Food Security = 1]	45.44	3.15	39.26	51.62	207.47	1	0.001*
[Food security = 2]	0^a^	.	.	.	.	.	.
BSE	1.11	0.22	0.67	1.55	24.60	1	0.001*
Susceptibility	0.034	0.13	−0.23	0.30	0.06	1	0.803
Threats	−0.013	0.16	−0.34	0.31	0.01	1	0.937
Barriers	−1.26	0.30	−1.85	−0.67	17.56	1	0.001*
Benefits	−0.67	0.34	−1.35	0.003	3.81	1	0.051
(Scale)	688.18						

^a^ Set at Zero because this parameter is redundant.

^b^ Maximum likelihood estimate.

* Significant at 0.05 Dependent variable: EBF days, Model: (Intercept), Time, Group, Income categorical, Household food security, BSES, Susceptibility, Threats, Barriers, and Benefits.

### 3.3 Discussion

Primary health care utilizes CHWs in the provision of basic services to people at the community level. The efforts are geared toward entrenching the community (level one) in the health delivery system, a strategy that provides a mechanism through which households and communities can take an active role in health‐related development issues. The community‐based approach is supposed to complement the formal health system.

The study therefore presents evidence‐based information for the implementation of community‐based EBF promotion strategies while strengthening already existing community‐based strategies for health promotion.

The aim of this study was to assess the effectiveness of CHWs in the promotion of EBF and EBI in the study area. The results of this study reveal effectiveness of CHWs in promoting EBF but not EBI. This study found the prevalence of EBI low, but consistent with the national rates according to KNBS & ICF Macro (2014) of 62% (<1 hr after birth). Similar findings were reported by Setegn et al. (2012) among mothers in Bale Goba District, South East Ethiopia where EBI prevalence was 52.4%. The rates also compare well with those of other East African countries like Uganda at 53% reported in the Uganda Demographic and Health Survey, (UBOS & ICF Macros, 2012). The above Country specific EBI rates were, however, not obtained in the context of interventions.

In our study, the rates reported are different from those obtained by Mbuya et al. (2019) who sought to increase rates of EBI and EBF by implementing an intervention delivered by village health workers that targeted context‐specific barriers. In their study, the prevalence of EBI was 86.6% in the intervention group verses 64.3% in the control group with a statistically significant difference.

Many factors have been reported to influence EBI. They include type of delivery assistance, mode of delivery, place of delivery; socio‐demographic and socio‐economic factors (KNBS & ICF Macro, 2014; John, Mistry, Kebede, Manohar, & Arora, 2019). In our study, two variables independently associated with EBI; not giving prelacteal feeds and place of delivery, (Mituki et al., 2017). It might mean that context‐specific barriers to EBI should be considered in the design of an intervention seeking EBI promotion.

The prevalence of EBF was also low at six months in the two groups in comparison with the national figures. The IG, however, had higher prevalence, but only statistically and significantly different at the 6th month.

It is worthwhile noting that clinic visits which provide an avenue for growth monitoring and EBF promotion for majority of Kenyan Mothers, with children below 9 months are modeled in the pattern of the National Immunization Schedule. The schedule consists of five contacts between birth and 9 months, (at birth, 6 weeks, 10 weeks, 14 weeks, and the 9th month). After the 14th week, up to the 9th month a breastfeeding mother may not visit the clinic unless the infant is unwell and conversely may not receive EBF promotion messages. This fact may be a potential reason why there were no statistically significant differences in EBF rates of mothers in the IG and CG, at 6, 10, and 14 weeks after adjusting for cluster randomization. Promotion of EBF at the Mother Child Health Clinics (MCH) may be effective but for as long as the mothers are attending these clinics. The difference was statistically significant at the 6th month. This difference may be attributable to the continuous contact of the mothers with CHWs when mothers are no longer attending MCH clinics in the IG and may be indicative of their effectiveness.

The study agrees with findings of Ochola et al. (2012). In an effort to determine the impact of home‐based intensive counseling strategies in the improvement of EBF rates in the Kibera slum in Nairobi Kenya, Ochola et al. (2012) reported a 23.6% EBF prevalence at 6 months in the home‐based intensive counseling group compared with 5.6% reported in the control. In yet another study by Kimani‐Murage et al. (2015) carried out in two slums of Nairobi and involving CHWs, mother–child pairs were followed longitudinally to establish EBF rates for the first 6 months. The control arm received usual care involving CHWs visits for counseling on antenatal and postnatal care, while the intervention arm received usual care and regular Maternal Infant and Young Child Nutrition (MIYCN) counseling by trained CHWs. Both the control and intervention groups received MIYCN information materials. The prevalence of EBF was 55% in the MIYCN‐Intervention group and 55% in the MIYCN‐Control. There were no statistically significant differences found in the two groups. This was linked to infiltration in the two groups. Nevertheless, the findings point out to the effectiveness of using CHWs in breastfeeding promotion. Similarly, Wangalwa et al. (2012) in a pre–post study that did not involve a control, investigated the effectiveness of CHWs in home‐based care to improve MCH outcomes. In this study, EBF rates improved from 20% to 50% postintervention.

Studies in different parts of Africa and the world have documented similar findings. For example in Ghana, a study that assessed the effectiveness of home‐based counseling in EBF reported a 40% rate in the intervention against 20% EBF rate in the control. Similarly, in a randomized controlled trial by Tylleskar et al. (2011), in three countries (Burkina Faso, Uganda, and South Africa) rates of EBF were higher in the intervention group in which CHWs were involved compared with the control (going through the regular healthcare services). In Burkina Faso, at 6 months rates of EBF were 71% and 9% for the intervention and control groups, respectively, in Uganda rates of EBF were 51% in the intervention group compared with 11% among the control participants. Similar results were observed in South Africa. In Brazil, Coutinho, Lira, Carvalho Lima, and Ashworth (2005) reported a huge effect in sustained EBF for up to 6 months for those mothers in the intervention which utilized the Baby Friendly Hospital Initiative (BFHI) combined with community support (45%) opposed to the group that utilized BFHI alone at 13%. These studies reveal that CHWs can offer breastfeeding support which can promote EBF for the first six months.

The study had strengths. First, we used cluster randomization which was successful since there were no statistically significant differences between the IG and CG groups in their baseline characteristics. Secondly, this was a longitudinal study and mother‐infant pairs were followed up to six months, the results therefore may reflect the true picture of rates of EBF since national data (from the Kenya Demographic Health Survey) uses a single 24‐hr recall cross‐sectionally to compute rates. The study, however, had several limitations, first, in the collection of EBI and EBF data, we relied on maternal recall, and there may have been courtesy bias which could have led to overestimating the effect of the intervention. To minimize this bias, we used research assistants who were blinded to the study group mothers belonged to collect data while the CHWs implemented the intervention. Secondly, there might have been infiltration since the proximity of the Health Centres and the different villages were close. To mitigate against this factor, analysis was adjusted for cluster randomization.

## 4 CONCLUSION AND RECOMMENDATIONS

This study revealed low rates of EBI and EBF among the mothers, the prevalence of EBF was, however, higher in the IG with a statistically significant difference than the CG at the 6th month. These results indicate that CHWs have potential effectiveness in promoting EBF. The Kenyan Governments move to roll out the Breastfeeding Community Initiative (BFCI) should be encouraged and adequately funded especially among the urban poor populations as it utilizes CHWs. This cadre of workers promises gains in improving EBF rates at the community level. Increased rates of EBF may contribute toward reduction in incidences of infant morbidities and mortalities, which are major concerns in the resource‐restricted areas. Further, it may aid in achieving the 3rd Sustainable Development Goal, (SDG), which aims to end preventable deaths of newborns and under fives by 2030. The study recommends that County Governments engage and empower CHWs especially in low‐resource settings, which are often underserved due to lack of access to healthcare services coupled with low socio‐economic status. This will ensure that mothers are followed up and offered support when dealing with challenges of EBF. This is particularly important for mothers after the 14th week postpartum, since mothers may not easily seek MCH services until the infant is 9 months.

Reorientation of Health Workers on EBI support and its importance once mothers deliver is also recommended. The authors also recommend that messages are tailored to emphasize the importance of EBI, discourage prelacteal feeds while encouraging hospital deliveries. Further it is recommended that context‐specific barriers to EBI be identified and intervened for.

## CONFLICT OF INTEREST

The authors declare that they have no competing interests.

## AUTHORS CONTRIBUTION

Mituki, Tuitoek, Varpolatai, and Ngotho were involved in the design development. Mituki was involved in data analysis and writing of the first draft of the manuscript. Mituki, Tuitoek, Varpolatai, and Ngotho contributed to writing of the manuscript. Mituki, Tuitoek, Varpolatai, Ngotho, and Kimani‐Murage reviewed the manuscript.

## ETHICAL STATEMENTS

Ethical clearance was obtained from Egerton University (Ref: EU/DVRE/028) and a research permit from the National Commission of Science Technology and Innovation of Kenya (NCST/RCD/12A/013/64). The trial was registered through the International Standard Randomized Controlled Trials Number (ISRCTN34314544) and in addition, permission sought from the study area District Ministry of Education office and from the Medical Officers at the Kiandutu and Makongeni Health Centres. Further informed signed consent was obtained from each respondent.

## INFORMED CONSENT

Written informed consent was obtained from all study participants.
